# Supplementary material for: Correlative Organelle Microscopy: Fluorescence Guided Volume Electron Microscopy of Intracellular Processes
Source: Front Cell Dev Biol. 2022 Apr 11;10:829545. doi: 10.3389/fcell.2022.829545 (PMC9035751; doi:10.3389/fcell.2022.829545)
Supplement: Supplementary file 6 [file DataSheet1.pdf]

Supplementary Material

1 Supplementary Figures

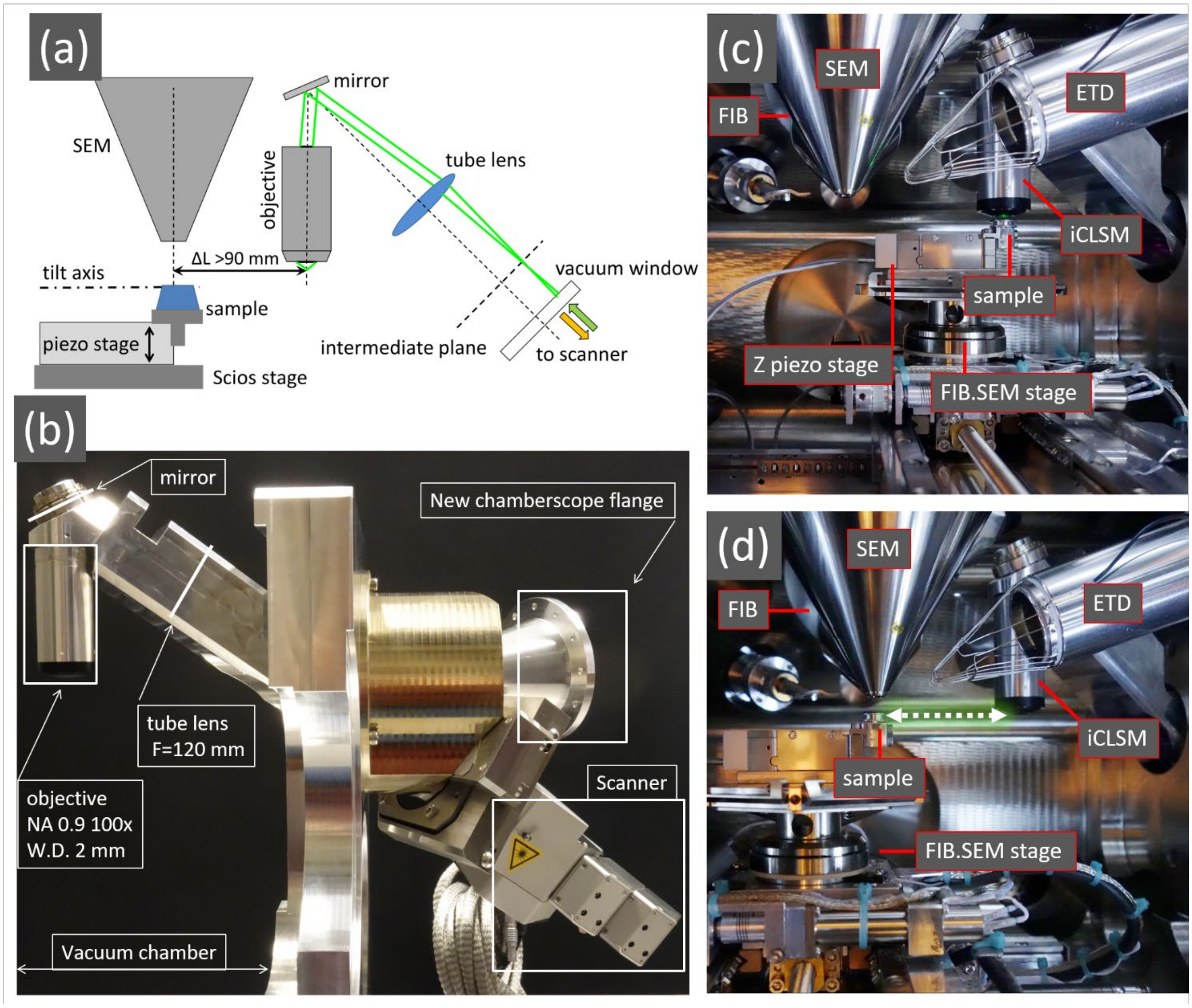

Supplementary Figure 1. The integrated CLSM- FIB.SEM setup. **(a)** Schematic of the integrated microscope. iCLSM and FIB.SEM observe the sample from the same direction. Samples can be investigated with the integrated CLSM by bringing the sample under the objective lens of the iCLSM. **(b)** iCLSM is built in a FEI Scios system, using a side entry port. Image shows the iCLSM as it is seen from the right wall of the vacuum chamber. **(c)(d)** Images of the FIB.SEM chamber with the mounted iCLSM set-up. Switching between iCLSM and (FIB.)SEM imaging is accomplished by stage translation using an accurate motorised stage of the FIB.SEM system.

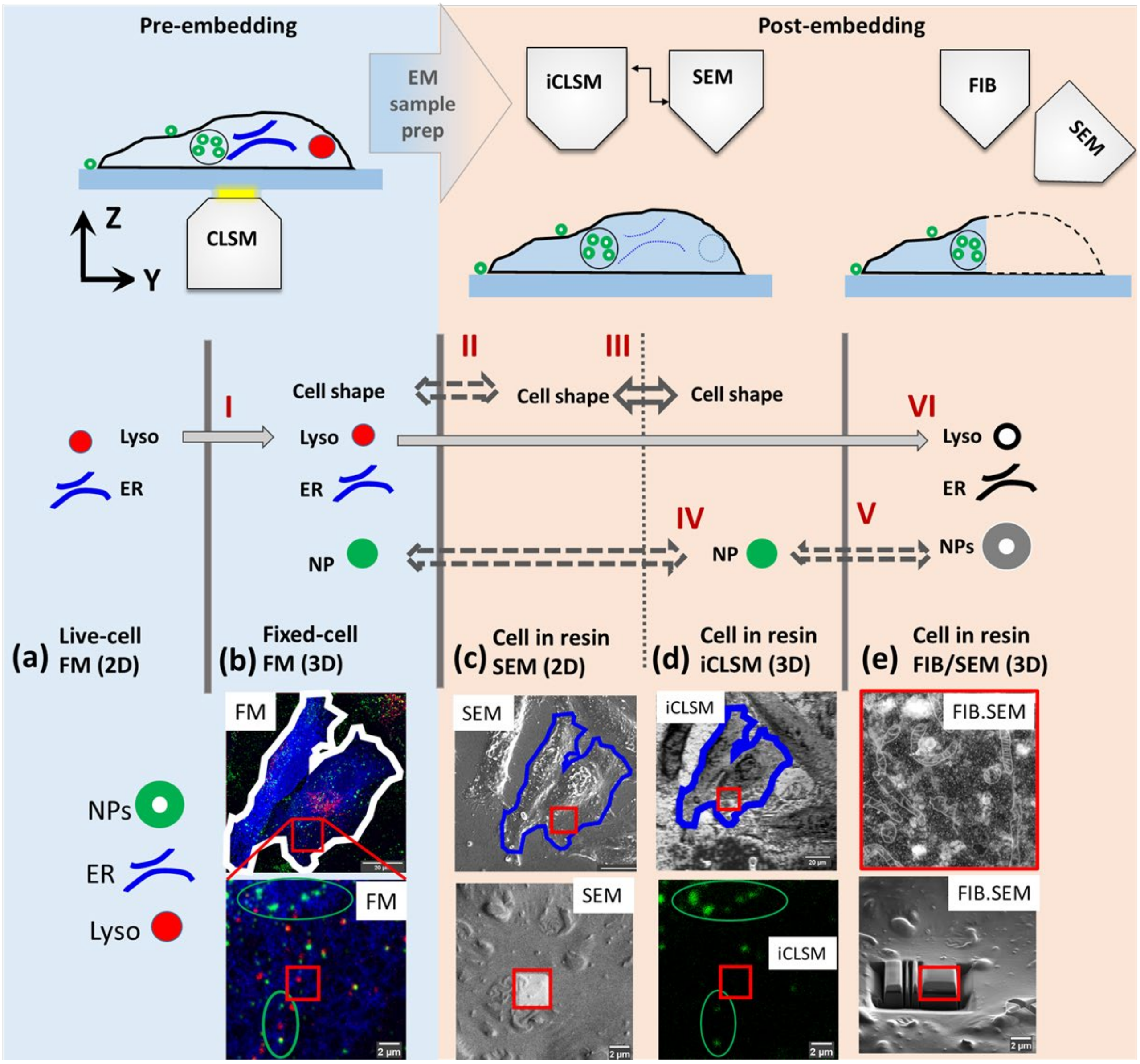

Supplementary Figure 2. Schematic showing the steps of correlative organelle microscopy workflow using integrated 3D CSLM and FIB.SEM. **(I)** Cells cultured on coverslips and labelled for distinct organelles are imaged in a CLSM, optionally first collecting (2D) live-cell videos, and then a (3D) z-stack of all channels, including fiducial nanoparticles (NP). **(II)** Cells are then prepared for EM by staining and resin embedding. Once placed in the integrated CLSM-FIB.SEM set-up, and SEM was used to localze the cells of interest and achieve a rough correlation with stand-alone CLSM. **(III)** The sample was shifted to iCLSM and an overview image was made with the reflection contrast. This step refines the ROI from the live-cell/fixed cell images (red square), and to determines the acquisition volumes of the FIB/SEM itself**. (iV)** iCLSM fluorescence channel then acquires the (3D) image of nano-fiducial particles in resin (green). **(V)** Next, the sample is translated back under the FIB.SEM. The ROI is selected in the SEM, prepared for FIB milling, and volumeEM data recorded with slice and view. **(VI)** Fiducial NPs are used for correlating FIB.SEM- iCLSM- CLSM datasets to each other. This generates an accurate translation matrix, and allows for correlation of each imaged organelle with high precision between 3D-FM and 3D-EM datasets.

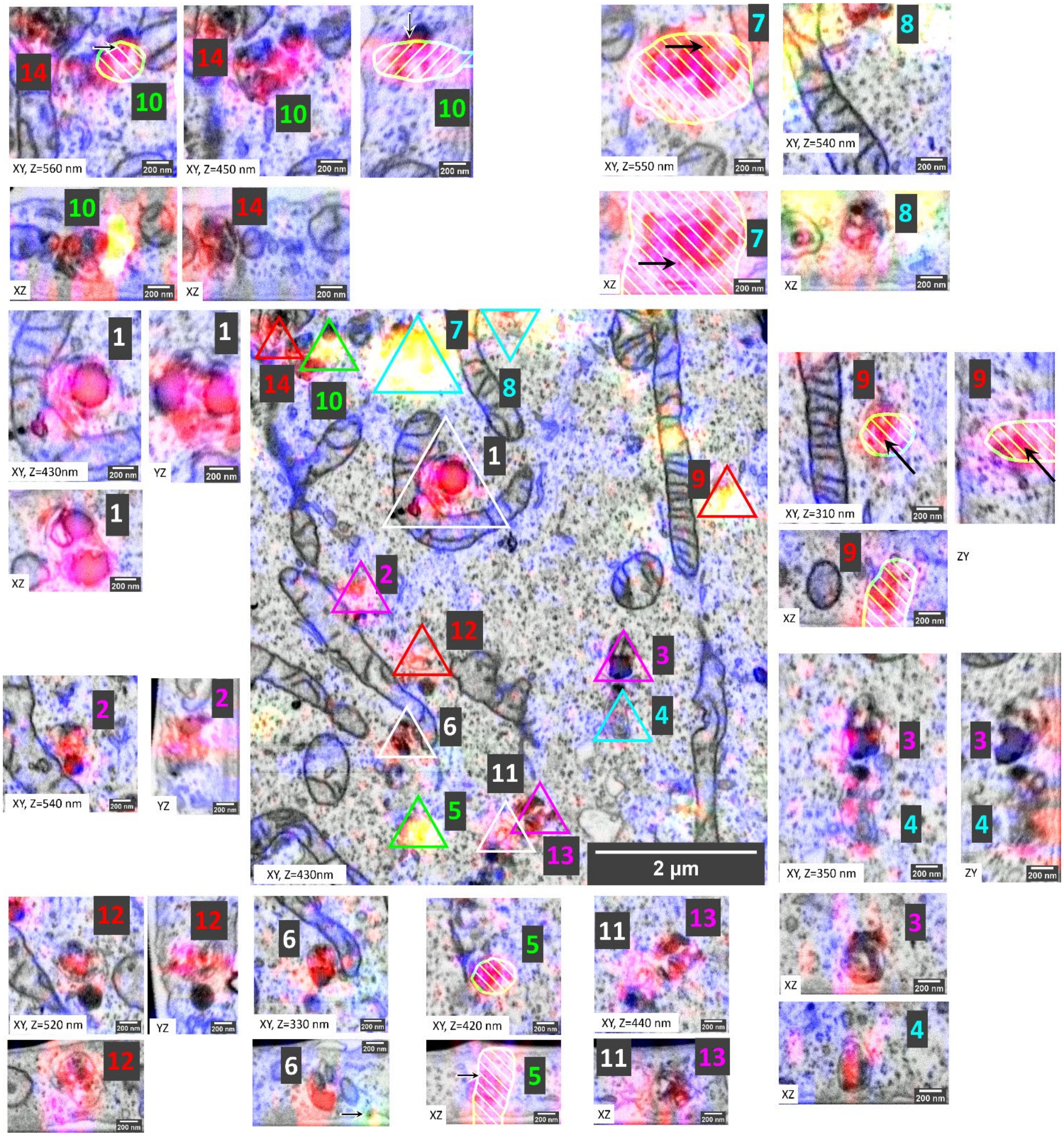

Supplementary Figure 3. Fluorescence data of every single live-cell imaged organelle in the ROI can be correlated to ultrastructural 3D-EM data with high precision. The cells have endocytosed fiducial particles (green), mEmerald-Sec61β is localized to ER (blue), and lysosomes are stained with SirLyso (red). Enzymatic activity of 14 lysosomes has been correlated to their ultrastructure.

Supplementary Information on Segmentation and Visualization

The segmentation program is implemented to segment a single organelle type in one run. If some of organelles were already segmented those voxels are suppressed in the present run (see the dark region in **Supplementary Figure 3**, corresponding to the previously segmented lysosome). The direction of slicing can be selected as XY or YZ or XZ slices. The best segmentation results are achieved when the segmentation is performed along all 3 possible directions. The results of these three runs is merged: a voxel is said to belong to the segmented organelle if it appears in any two of the three segmentation runs.

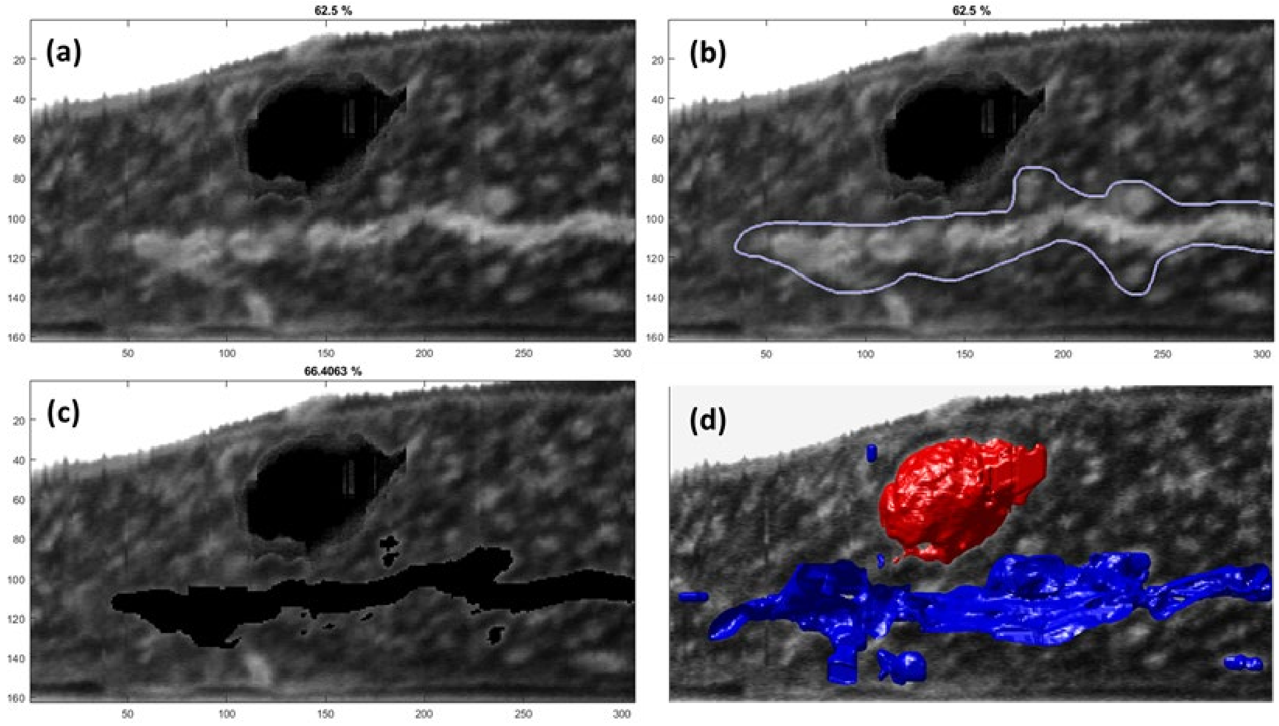

Supplementary Figure 4. (a) The average intensity of 10 XZ slices of a 3D Stack are presented to the user. The dark area at the top part of the cell is a lysosome segmented at the previous segmentation stage. **(b)** The area where the ER is predicted is selected by the user and highlighted with blue line. **(c)** Automatic segmentation was performed over the manually selected area on all of the slices of the image bunch. The pixels selected are set to zero. The user can now define a new area for segmentation on the same bunch or process to the next bunch of slices. **(d)** Segmented organelles are visualized in 3D.

The user is presented with averaged 10 consecutive 2D slices (i.e. bunched slices) in a full 3D stack. The manually selected area where the organelle of interest is visible is then subjected to automated segmentation. Each of the slices of the bunch is processed automatically by applying intensity threshold and edge detection. Next, the image of the same bunch (excluding just segmented areas) is shown again to the user to select another piece of the organelle. When there are no more non-segmented areas remain in the current bunch of slices, the user can double click) to proceed to the next bunch.

The program was implemented in MatLab with Image Processing Toolbox and Parallel Computing Toolbox. The extended code can be found at: https://github.com/UUtrechTT/Slice_and_View_postprocessing_code_MatLab

2 Supplementary Videos

1. Supplementary 3D-overlay Video 1 (related to Figure 1i &j and Figure 2d)
2. Supplementary live-cell Video 2 (related to Figure 2a)
3. Supplementary 3D-overlay Video 3 (related to Figure 3d)
4. Supplementary live-cell Video 4 (related to Figure 4a)
5. Supplementary 3D-overlay Video 5 (related to Figure 4d):
6. Supplementary 3D-segmentation Video 6 (related to Figure 4e)
7. Supplementary 3D-segmentation Video 7 (related to Figure 4e)
8. Supplementary live-cell Video 8 (related to Figure 5a and 5b)
9. Supplementary 3D-overlay Video 9 (related to Figure 5c)
10. Supplementary 3D-segmentation Video 10 (related to Figure 5c)
11. Supplementary 3D-segmentation Video 11 (related to Figure 5c)
12. Supplementary 3D-overlay Video 12 (related to Figure 5d)
13. Supplementary 3D-segmentation Video 13 (related to Figure 5d)
14. Supplementary 3D-overlay Video 14 (related to Figure 5e)
15. Supplementary 3D-segmentation Video 15 (related to Figure 5e)
